# Time Interval Between Right Ventricular Early Diastolic Velocity by Tissue and Pulse Wave Doppler: An Index of Right Atrial Pressure in Pulmonary Hypertension Patients

**DOI:** 10.3390/jcm13216349

**Published:** 2024-10-23

**Authors:** Costanza Natalia Julia Colombo, Francesco Corradi, Valentino Dammassa, Davide Colombo, Alessandro Fasolino, Mauro Acquaro, Susanna Price, Stefano Ghio, Guido Tavazzi

**Affiliations:** 1 Anesthesia and Intensive Care, Fondazione IRCCS Policlinico San Matteo, 27100 Pavia, Italy; 2 PhD Program in Experimental Medicine, University of Pavia, 27100 Pavia, Italy; 3 Department of Surgical, Medical and Molecular Pathology and Critical Care Medicine, University of Pisa, 56126 Pisa, Italy; 4 Adult Intensive Care Unit, Royal Brompton Hospital, London SW3 6NP, UK; 5 Division of Cardiology, Fondazione IRCCS Policlinico S. Matteo, 27100 Pavia, Italy; 6 National Heart and Lung Institute, Imperial College, London SW7 2AZ, UK; 7 Department of Clinical-Surgical, Diagnostic and Pediatric Sciences, University of Pavia, 27100 Pavia, Italy

**Keywords:** time intervals, early diastolic velocity, right atrial pressure, Doppler, pre-capillary pulmonary hypertension

## Abstract

**Background:** A reversal of time difference between the onset of early diastolic velocity (e’) during tissue Doppler imaging and the onset of mitral inflow (E) has been observed in cases of elevated left atrial pressure. Whether this interval (T_e’-E_) may be useful to assess right atrial pressure has never been investigated, neither in healthy subjects nor in pulmonary hypertension patients. **Methods:** Right ventricular T_e’-E_ was assessed in patients with pre-capillary pulmonary hypertension and compared with healthy volunteers who underwent comprehensive echocardiography examination. T_e’-E_ is the difference between the interval from R wave at the superimposed electrocardiogram to the e’ wave during right ventricular tissue Doppler imaging and the interval from the R wave to transtricuspid E wave during pulsed wave Doppler imaging. Right atrial pressure was invasively measured in pulmonary hypertension patients. **Results:** Fifty-six patients were enrolled. T_e’-E_ was prolonged in pulmonary hypertension subjects compared with healthy subjects (*p* < 0.001). Amongst the pulmonary hypertension patients, strong correlations were found between T_e’-E_ and right atrial pressure (r = −0.885, *p* < 0.001), systolic pulmonary pressure (r = −0.85, *p* < 0.001) and the duration of tricuspid regurgitation (r = 0.72, *p* < 0.001). The area under the receiver operating characteristic curve of T_e’-E_ in identifying right atrial pressure higher than 15 mm of mercury was 0.992 (sensitivity 100%, specificity 83%). **Conclusions:** In contrast to the left ventricle, there is a delay in the proto-diastolic filling in pulmonary hypertension patients, which correlates with the increase in systolic pulmonary arterial pressure, right atrial pressure, tricuspid regurgitation duration and restrictive diastolic pattern.

## 1. Introduction

Right atrial pressure (RAP) is a key parameter usually evaluated in critically ill and cardiovascular populations and it has been described as a prognostic marker in patients with pulmonary hypertension and acute heart failure [1]. Although the gold standard method to assess RAP is the invasive measure of this parameter, this requires either the placement of a central venous line or a pulmonary Swan-Ganz catheter. Alternatively, the non-invasive method using echocardiography to evaluate the static inferior vena cava diameter plus its variation during respiratory phases is widely applied [2].

However, the non-invasive assessment has several established limitations, including right ventricular (RV) dysfunction, moderate or severe tricuspid regurgitation, abdominal compartment syndrome, alterations in heart lung interaction, presence of mechanical support cannula and significant respiratory efforts producing markedly negative intrathoracic pressures. Furthermore, inferior vena cava dimensions and derived indices can identify an increased RAP, but they may fail in estimating the magnitude of RAP elevation [3,4].

The time interval between the onset of early diastolic velocity during tissue Doppler imaging (e’ wave) and the onset of mitral inflow (E wave) has proved to be a sensitive index to assess the presence of high left atrial pressure in patients with valvular disease (both in the case of mitral regurgitation and mitral stenosis) [5]. Physiologically, the tissue velocity displacement (e’ wave) occurs a few milliseconds earlier than the early diastolic flow entering from the atrium to the ventricular chamber (E wave). In cases of severely increased left atrial pressure, a reversal of this relation has been observed, with the mitral inflow being the first wave detected. This happens because in cases of impaired relaxation and pseudonormal LV filling, the left ventricular (LV) e’ wave is significantly delayed.

The total isovolumic time (t IVT) represents the time (expressed in second/minute) when the ventricles do not eject nor fill, therefore describing the total amount of isovolumic phases in the cardiac cycle, adjusted by the heart rate. It is an accurate marker of ventricular efficiency and systo-diastolic interaction. Left-sided t IVT prolongation has been strongly associated with the presence of ischemic cardiac disease and heart failure [6]. However, there are no validation studies that have established the role of right ventricular t IVT (RV t IVT) in populations with different cardiovascular diseases.

In pulmonary hypertension (PH), amongst all the pathophysiological modifications in RV function including the increased RAP, there is also diastolic dysfunction due to the RV stiffness in response to the increased load [7]. The pulmonary valve pre-ejection wave (PV A wave) is an index of the right ventricular diastolic restrictive pattern. It can be detected as an anterograde end-diastolic pulmonary arterial flow coincident with the premature opening of the pulmonary valve during the right atrial systole, determined by the equalization of the RV end-diastolic pressure and the pulmonary arterial diastolic pressure [8].

Whether the time interval between the onset of RV diastolic myocardial relaxation and early tricuspid inflow may be useful to assess RAP has never been investigated, neither in healthy subject nor in patients with diseases leading to cardio-pulmonary pathophysiological modifications. Additionally, the clinical relevance of increased RAP and its association with RV systo-diastolic interaction and diastolic compliance have never been assessed.

We sought to measure the time interval between the RV lateral wall e’ and tricuspid E wave (RV T_e’-E_) in healthy subjects and patients with pre-capillary pulmonary hypertension, assessing any correlation with RAP, RV t IVT and the presence of the PV A wave.

## 2. Materials and Methods

### 2.1. Echocardiography

The study population was composed of a matched cohort of patients with pre-capillary PH and healthy volunteers from the RIVITA study [9]. The study was approved by the local ethical committee and patients signed informed consent for the treatment and use of data for scientific purposes. On top of the standard echocardiographic study performed in accordance with the most recent guideline recommendations [10], we measured RV time intervals using a simultaneous electrocardiogram (ECG) displayed on the echocardiographic system and averaged three consecutive measurements for each parameter. The frame rate was 100 mm/s. All the echocardiographic data were acquired at the end of expiration.

The E wave was detected with pulsed wave Doppler imaging with the sample volume at the tip of the tricuspid leaflets in parasternal long-axis view of RV inflow or parasternal short-axis view; the e’ wave was collected with tissue Doppler imaging in apical four-chamber view, placing the sample volume on the basal segment of the RV free wall.

**Figure 1:**
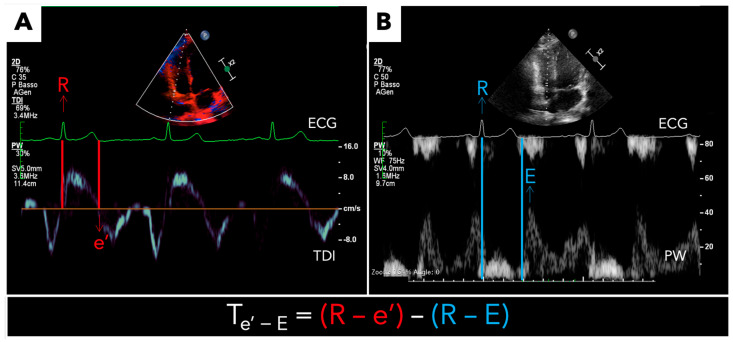
**Right ventricular R-e’ and R-E measurements.** (**A**) R-e’ wave time interval (red): time from R wave during superimposed ECG to the onset of e’ during RV tissue Doppler imaging; (**B**) R-E wave time interval (light blue): time from R wave during superimposed ECG to the onset of E during transtricuspid pulse wave Doppler imaging. T_e’-E_ = [(R-e’ time interval) − (R-E time interval)]).

RV T_e’-E_ was calculated as the difference between the time from the R wave during the superimposed ECG to the onset of e’ during RV tissue Doppler imaging (Figure 1A) and the interval from the R wave during superimposed ECG to the onset of E during transtricuspid pulse wave Doppler imaging (Figure 1B), as shown in the following equation: T_e’-E_ = (R-e’) − (R-E).

In healthy subjects, RAP was assessed non-invasively, derived from echocardiographic parameters. In PH patients, RAP was measured by invasive central vein catheter and systolic pulmonary arterial pressure was derived from tricuspid regurgitation velocity, applying the modified Bernoulli equation. RV t IVT was also captured. Right ventricular filling time (RV FT) was measured as the total duration of trans-tricuspid inflow evaluated placing the sample volume at the tip of the tricuspid leaflets in parasternal long-axis view of RV inflow or parasternal short-axis view. RV ejection time (RV ET) was measured as the time interval from the onset of forward pulmonary flow to the pulmonary valve closure artifact in parasternal long-axis view of RV outflow tract or parasternal short-axis view, placing the sample volume on the pulmonary valve.

**Figure 2:**
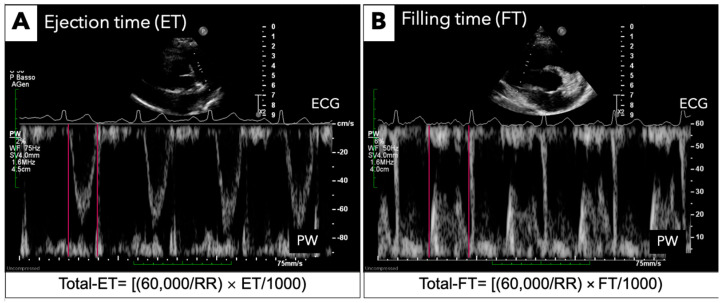
**Total isovolumic time echocardiographic measurement.** (**A**) RV total ejection time; (**B**) RV total filling time. Total isovolumic time is measured in seconds/minute.

RV total ejection time (t ET) and total filling time (t FT), expressed as seconds, were derived as the product of the corresponding time interval and heart rate, using the following formulae: t ET = [(60,000/RR) × RV ET]/1000 (Figure 2A) and t FT = [(60,000/RR) × RV FT]/1000. RV t IVT (Figure 2B), measured in seconds/minute, was calculated as the difference between 60 s and the sum of t ET and t FT (t IVT = 60 − [t FT + t ET]), previously derived. T ET and t FT were obtained using the RR interval of the respective cardiac cycle [9,10].

The presence of the PV A wave was assessed detecting an anterograde flow through the pulmonary valve in correspondence to the atrial systole (P wave during the superimposed ECG) [8].

All the procedures were followed in accordance with the ethical standards of the responsible committee on human experimentation and with the Declaration of Helsinki.

### 2.2. Statistical Analysis

IBM^®^ SPSS version 27 Statistics was used for data computation. Normal distribution of data was assessed both with the D’Agostino–Pearson test and histogram representation. Data with normal distribution are expressed as mean with standard deviation (SD); otherwise, they are presented as the median value and interquartile range (IQR). Echocardiographic parameters were compared using Student’s *t*-test; a *p*-value < 0.05 was considered statistically significant. Correlations were assessed for each group (healthy subjects and PH patients) with Pearson analysis and definition of r coefficients. The levels of agreement were considered according to the literature, as follows: poor < 0.40; fair 0.40–0.60; good 0.60–0.75 and excellent 0.75–1 [11]. Receiver operating characteristic (ROC) analyses were also performed to test the performance of the RV T_e’-E_ interval in detecting elevated RAP.

## 3. Results

In total, 56 patients were enrolled: 28 were healthy subjects and 28 had a pre-capillary PH diagnosis, according to the current guidelines. The two groups were similar in terms of age (55.9 ± 9.8 and 55.9 ± 9.7 years old, respectively; *p* = 0.996) and sex distribution (67% of the healthy subjects were female and 65% in the PH group; *p* = 0.95).

The mean values of RAP, echocardiographic parameters, and derived time intervals in the two groups are presented in Table 1.

Despite comparable RR intervals (*p* = 0.442), patients with PH presented longer R-E and R-e’ time intervals compared to healthy subjects (*p* < 0.001). In addition, R-E was significantly longer than R-e’ (*p* < 0.001) in PH patients, but not in healthy volunteers, who had almost no difference between these two time intervals. Consequently, the RV T_e’-E_ length was significantly longer between healthy volunteers and PH patients (*p* < 0.001).

The IVC diameter was not significantly different between the two groups, while IVC inspiratory collapse was more frequent in cases of PH. In reference to RV systolic function, the echocardiographic data collected amongst PH patients showed a systolic impairment, with significantly lower tricuspid annular systolic excursion (TAPSE) and systolic wave peak during tissue Doppler imaging; PH patients also had a significant RV dilatation, considering the RV diameter. Considering RV systo-diastolic interaction, a significantly longer RV t IVT was observed amongst PH patients.

**Figure 3:**
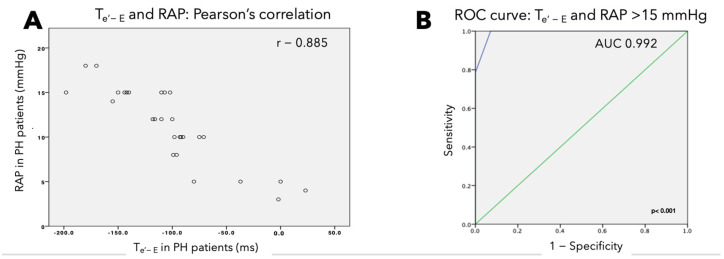
(**A**) Scatter plot showing correlation between T_e’-E_ and RAP; (**B**) area under the receiver operating characteristic curve of the time interval between e’ and E in predicting right atrial pressure.

In PH patients, strong correlations between RAP and RV T_e’-E_ (r = −0.885, *p* < 0.001) (Figure 3A) and between systolic pulmonary arterial pressure and RV T_e’-E_ (r = −0.85, *p* < 0.001) were found.

A negative correlation between RV T_e’-E_ and the presence of PV A wave (r = −0.76, *p* < 0.001) was found, as well RV T_e’-E_ and tricuspid regurgitation duration (r = −0.72, *p* < 0.001).

Furthermore, amongst PH patients, a positive association between RAP and RV t IVT (r = 0.584, *p* < 0.01) was also demonstrated; conversely, the E/e’ ratio was weakly correlated with RAP and systolic pulmonary arterial pressure (r = 0.28, *p* = 0.03 and r = 0.21, *p* = 0.04, respectively).

The area under the ROC curve of RV T_e’-E_ in identifying RAP higher than 15 mm of mercury (mmHg) (Figure 3B) was 0.992 (sensitivity: 100%, specificity: 83%, positive predictive value: 100%, negative predictive value: 73%) whereas the ROC for E/e’ ratio carried low accuracy (sensibility: 56% and specificity: 18%).

## 4. Discussion

The main result of the study is a significant correlation between the increased invasively measured RAP and the prolongation of the time difference between the myocardial activation and the proto-diastolic filling in PH patients.

Additionally, a significant and remarkable association was found with the prolongation of RV t IVT, the increase in systolic pulmonary arterial pressure and tricuspid regurgitation duration and restrictive RV diastolic pattern (detection of PV A wave).

Previous studies that focused on cardiovascular diseases affecting the left ventricle have shown that when LV long-axis expansion and relaxation are delayed, such as in LV heart failure and the coronary artery stenosis model, e’ during tissue Doppler imaging is prolonged, thus occurring after the onset of mitral inflow [12]. The underlying pathophysiological mechanism of this phenomenon consists of the high left atrial pressure acting as the main driver of the early opening of the mitral valve and thus early onset of LV diastolic filling, instead of myocardial relaxation.

In normally functioning RV, as shown in healthy subjects, the tricuspid inflow (E wave) and tissue activation (e’) start almost contemporarily with an isovolumic relaxation time ranging from 2 to 15 ms [9]. The normal RV pressure–volume relationship is characterized by a trapezoidal shape, due to the high efficiency/low impedance pulmonary circulation. In cases of pulmonary hypertension, the RV pressure–volume loop changes from its typical shape to a square or rectangular one, with prolonged isovolumic contraction and relaxation periods, resembling the left ventricle loop [13]. This reflects the adaptation of time intervals according to the RV function and morphological remodeling [14]. A clear association between isovolumetric times and prognosis in pulmonary hypertension patients has been demonstrated [15]. It is probable that the prolongation of isovolumetric times has direct consequences on the RV hemodynamic and interventricular dependence. Due to the prolonged isovolumetric contraction of the RV, early diastolic LV filling is hampered. The main mechanism appears to be increased wall tension [16]: RV contraction continues while the LV is already in its diastolic phase. Because of the leftward shift of the septum, the pulmonary valve will close while the RV is still contracting. This is called “post-systolic isovolumetric contraction”, which contributes to the mechanical inefficiency because the energy of the contraction in that phase in the cardiac cycle is not used to deliver forward flow. The prolongation of post-systolic isovolumetric time in cases of pulmonary hypertension, measured by means of Doppler echocardiography [17], should be considered as a measure of disease severity; in fact, amongst PH patients, an association with this prolongation with pulmonary vascular resistance, PAPs and RV ejection fraction was observed [7,18].

In our study, the prolongation of T_e’-E_ (from almost 0 ms in healthy volunteers to an absolute value of 100 ms in pulmonary hypertension patients) may be justified by different mechanisms. The prolongation of isovolumetric times reflects the time needed by the RV to gain energy to overcome the afterload to open pulmonary valve and deliver perfusion flow. This prolongation leads to a delay in tricuspid valve opening. Additionally, the presence of significant tricuspid regurgitation may delay and shorten the RV filling. Although there is no cut off value of clinically significant length of tricuspid regurgitation, a cardiac magnetic resonance study demonstrated that a duration of tricuspid regurgitation > 400 ms was associated with RV function and events such as cardiovascular mortality and hospital readmission [19].

The relation between the systolic and diastolic times has been investigated and validated using the myocardial performance index in pulmonary hypertension populations. The myocardial performance index integrates the isovolumetric times by subtracting the ejection time from the interval between the end of filling period to the onset of the following one as the numerator [20]; the result is then divided by the ejection time. The myocardial performance index does capture the variation in the systo-diastolic interaction and it has been validated as a prognostic factor in heart failure and pulmonary hypertension patients. However, LV t IVT has shown to be superior to the myocardial performance index in depicting the LV activation and performance due to some flaws, as the myocardial performance index does not include ejection time in the numerator or denominator in its formula, and it is not adjusted by the heart rate [21].

RV t IVT is a sensitive marker of systo-diastolic interaction. In fact, considering its formula, this parameter integrates in a single value both the diastolic and systolic phase of the cardiac cycle. The normal value is 7 ± 1.1 s/min and varies significantly with increasing age, from a minimum of 3.4 s/min to a maximum of 9.7 s/min [9]. t IVT measurement also includes the filling time, which is directly affected by ischemic processes and the severity of valvular regurgitation. RV t IVT prolongation reflects the extension of isovolumetric times, which could be due to either afterload increase or ischemic processes, therefore reflecting a reduction in RV performance. Formerly validated for the left ventricle in dilated cardiomyopathy, post-cardiotomic and cardiogenic shock patients, t IVT seemed to be amongst the most accurate parameter of LV performance [6,22,23,24]. Up to date, no data exist on the role of RV t IVT in cardiovascular diseases and pulmonary hypertension.

PV A wave was originally described in the congenital population and reported in the presence of RV diastolic pressure above 20 mmHg, as an index of restrictive diastolic compliance [25]. It occurs when the RV becomes unable to stretch after proto-diastolic filling leading to premature pulmonary valve opening and thus to anterograde flow during atrial contraction [25,26]. It is highly reproducible, and it does not require additional views on the pulmonary valve ejection, which is routinely acquired to evaluate either pulmonary valve acceleration time, RV velocity time integral shape and related measurements. Nevertheless, PV A wave is mostly neglected in the pulmonary hypertension population and never reported for the RV diastolic assessment.

RV diastolic assessment relies on a few parameters, such as the E/A ratio, E/e’ ratio, suprahepatic vein flow pattern and inferior vena cava, without a strict categorization as for the LV.

The E/A ratio reduces with increasing age and has never been validated under different loading conditions; an E/e’ ratio < 6 is considered normal. However, this value is derived by only two studies. In the first study, an E/e’ ratio ≥ 4 was associated with an RAP > 10 mmHg [27], whereas in the second study, this association was found for an E/e’ ratio > 8 [28]. In all these studies, pulmonary pressures were not significantly elevated, therefore questioning the applicability of such cut-off values at different loading conditions or in cases of PH. Additionally, a previous study with a pressure–volume loop analysis in pulmonary hypertension patients demonstrated that tricuspid E/A and E/e′ ratios did not correlate with RV end-diastolic elastance [29]. Suprahepatic vein flow waveform variation may be related to different conditions, including RV systolic dysfunction, pulmonary pressures, respiratory and liver diseases [30]. Furthermore, the inferior vena cava measurement may be affected by several conditions [3], and thus may be unreliable in the assessment of RV diastolic dysfunction.

### Limitations

We acknowledge some limitations of our study. The PH population in the current study is characterized by patients with significant RV systolic dysfunction and moderate to severe estimated pulmonary pressure. Therefore, these results should be contextualized, and further validation is needed both in a larger setting and in patients with normal RV function.

The major limitation of this study is the small sample size and the lack of validation of the results with a gold standard monitoring device, such as a pressure–volume loop analysis.

## 5. Conclusions

In pulmonary hypertension patients, RV T_e’-E_ prolongation was associated with high RAP, reflecting a significant delay in protodiastolic RV filling; significant correlations between this time prolongation and systolic pulmonary arterial pressure, tricuspid regurgitation duration and restrictive RV diastolic pattern were also observed. These data need to be validated in a wider population with pulmonary hypertension from different etiologies.

## Figures and Tables

**Table 1:** Description of population features.

Parameter	Healthy Subjects	Pulmonary Hypertension Patients	*p* Value
sPAP, mmHg	-	64.33 ± 14.58	N/A
TAPSE, mm	25.82 ± 3.73	12.5 ± 2.64	<0.001
RV annular diameter, mm	28.68 ± 4.76	57.48 ± 5.27	<0.001
RAP, mmHg	3.43 ± 1.07	13.29 ± 4.22	<0.001
RV E/e’	4.19 ± 1.07	8.66 ± 2.7	0.001
RV s’, cm/s	12.60 (11.9–18.65)	10.65 (9.38–12.00)	<0.001
RV e’, cm/s	9.37 ± 2.25	7.47 ± 1.67	0.001
RR interval, ms	721.36 ± 221.35	754.82 ± 56.16	0.442
TR duration, ms	-	535 (520–580)	N/A
IVC diameter, mm	12.56 ± 2.77	14.2 ± 3.83	0.342
IVC inspiratory collapse, %	28	7	<0.001
RV t FT, s/min	29.39 ± 9.04	29.85 ± 8.85	<0.01
RV t ET, s/min	25.96 ± 2.42	14.92 ± 1.80	<0.001
RV t IVT, s/min	6 ± 3.43	13 ± 4.43	<0.001
R-E wave time interval, ms	303.89 ± 113.20	566.39 ± 39.13	<0.001
R-e’ wave time interval, ms	303.21 ± 112.63	464.54 ± 29.86	<0.001
T_e’-E_ time interval, ms	0.036 ± 11.846	−101.857 ± 52.09	<0.001

sPAP—systolic pulmonary arterial pressure; mmHg—millimeters of mercury; TAPSE—tricuspid annular systolic excursion; RV—right ventricular; RAP—right atrial pressure; RV E/e’—ratio between E and e’ wave; RV s’—systolic wave peak at TDI; TR—tricuspid valve regurgitation; IVC—inferior vena cava; t FT—total filling time; t ET—total ejection time; t IVT—total isovolumic time; R-E—time interval from R wave during superimposed ECG to the onset of E during transtricuspid pulse wave Doppler imaging; R-e’—time interval from R wave during superimposed ECG to the onset of e’ during RV tissue Doppler imaging; T_e’-E_—time interval between RV lateral wall e’ and tricuspid E wave.

## Data Availability

Data are available upon request from the authors.

## References

[1] Cogswell R., Pritzker M., Marco T.D. (2014). Performance of the REVEAL Pulmonary Arterial Hypertension Prediction Model Using Non-Invasive and Routinely Measured Parameters. J. Heart Lung Transplant..

[2] Beigel R., Cercek B., Luo H., Siegel R.J. (2013). Noninvasive Evaluation of Right Atrial Pressure. J. Am. Soc. Echocardiogr..

[3] Via G., Tavazzi G., Price S. (2016). Ten Situations Where Inferior Vena Cava Ultrasound May Fail to Accurately Predict Fluid Responsiveness: A Physiologically Based Point of View. Intensive Care Med..

[4] Tavazzi G., Alviar C.L., Vandenbriele C., Corradi F. (2024). Right Ventricular Diastolic Dysfunction and Venous Pulsatile Pattern: A Manifestation of Heart-Lung Interactions in Mechanical Ventilation?. CHEST.

[5] Diwan A., Diwan A., McCulloch M., Lawrie G.M., Reardon M.J., Nagueh S.F. (2005). Doppler Estimation of Left Ventricular Filling Pressures in Patients with Mitral Valve Disease. Circulation.

[6] Duncan A.M., Francis D.P., Henein M.Y., Gibson D.G. (2003). Limitation of Cardiac Output by Total Isovolumic Time during Pharmacologic Stress in Patients with Dilated Cardiomyopathy: Activation-Mediated Effects of Leftbundle Branch Block and Coronary Artery Disease. J. Am. Coll. Cardiol..

[7] Noordegraaf A.V., Westerhof B.E., Westerhof N. (2017). The Relationship Between the Right Ventricle and Its Load in Pulmonary Hypertension. J. Am. Coll. Cardiol..

[8] Tavazzi G., Bergsland N., Alcada J., Price S. (2019). Early Signs of Right Ventricular Systolic and Diastolic Dysfunction in Acute Severe Respiratory Failure: The Importance of Diastolic Restrictive Pattern. Eur. Heart J. Acute Cardiovasc. Care.

[9] Tavazzi G., Boffi A., Savioli G., Greco A., Pavesi C., Klersy C., Guida S., Iotti G., Mojoli F., Ghio S. (2019). Right Ventricular Total Isovolumic Time: Reference Value Study. Echocardiography.

[10] Rudski L.G., Lai W.W., Afilalo J., Hua L., Handschumacher M.D., Chandrasekaran K., Solomon S.D., Louie E.K., Schiller N.B. (2010). Guidelines for the Echocardiographic Assessment of the Right Heart in Adults: A Report from the American Society of Echocardiography Endorsed by the European Association of Echocardiography, a Registered Branch of the European Society of Cardiology, and the Canadian Society of Echocardiography. J. Am. Soc. Echocardiogr..

[11] Liljequist D., Elfving B., Roaldsen K.S. (2019). Intraclass Correlation—A Discussion and Demonstration of Basic Features. PLoS ONE.

[12] Rivas-Gotz C., Khoury D.S., Manolios M., Rao L., Kopelen H.A., Nagueh S.F. (2003). Time Interval between Onset of Mitral Inflow and Onset of Early Diastolic Velocity by Tissue Doppler: A Novel Index of Left Ventricular Relaxation Experimental Studies and Clinical Application. J. Am. Coll. Cardiol..

[13] Friedberg M.K., Redington A.N. (2014). Right Versus Left Ventricular Failure. Circulation.

[14] Dambrauskaite V., Delcroix M., Claus P., Herbots L., Palecek T., D’hooge J., Bijnens B., Rademakers F., Sutherland G.R. (2005). The Evaluation of Pulmonary Hypertension Using Right Ventricular Myocardial Isovolumic Relaxation Time. J. Am. Soc. Echocardiogr..

[15] Gerges M., Gerges C., Pistritto A.-M., Lang M.B., Trip P., Jakowitsch J., Binder T., Lang I.M. (2015). Pulmonary Hypertension in Heart Failure. Epidemiology, Right Ventricular Function, and Survival. Am. J. Respir. Crit. Care Med..

[16] Gan C.T.-J., Lankhaar J.-W., Marcus J.T., Westerhof N., Marques K.M., Bronzwaer J.G.F., Boonstra A., Postmus P.E., Vonk-Noordegraaf A. (2006). Impaired Left Ventricular Filling Due to Right-to-Left Ventricular Interaction in Patients with Pulmonary Arterial Hypertension. Am. J. Physiol.-Heart Circ. Physiol..

[17] Tei C. (1995). New Non-Invasive Index for Combined Systolic and Diastolic Ventricular Function. J. Cardiol..

[18] Mauritz G.-J., Marcus J.T., Westerhof N., Postmus P.E., Vonk-Noordegraaf A. (2011). Prolonged Right Ventricular Post-Systolic Isovolumic Period in Pulmonary Arterial Hypertension Is Not a Reflection of Diastolic Dysfunction. Heart.

[19] Cho I.-J., Oh J., Chang H.-J., Park J., Kang K.-W., Kim Y.-J., Choi B.-W., Shin S., Shim C.Y., Hong G.-R. (2014). Tricuspid Regurgitation Duration Correlates with Cardiovascular Magnetic Resonance-Derived Right Ventricular Ejection Fraction and Predict Prognosis in Patients with Pulmonary Arterial Hypertension. Eur. Heart J. Cardiovasc. Imaging.

[20] Tei C., Ling L.H., Hodge D.O., Bailey K.R., Oh J.K., Rodeheffer R.J., Tajik A.J., Seward J.B. (1995). New Index of Combined Systolic and Diastolic Myocardial Performance: A Simple and Reproducible Measure of Cardiac Function—A Study in Normals and Dilated Cardiomyopathy. J. Cardiol..

[21] Duncan A.M., Francis D.P., Henein M.Y., Gibson D.G. (2004). Importance of Left Ventricular Activation in Determining Myocardial Performance (Tei) Index: Comparison with Total Isovolumic Time. Int. J. Cardiol..

[22] Tavazzi G., Kontogeorgis A., Bergsland N.P., Price S. (2016). Resolution of Cardiogenic Shock Using Echocardiography-Guided Pacing Optimization in Intensive Care. Crit. Care Med..

[23] Tavazzi G., Kontogeorgis A., Guarracino F., Bergsland N., Martinez-Naharro A., Pepper J., Price S. (2017). Heart Rate Modification of Cardiac Output Following Cardiac Surgery. Crit. Care Med..

[24] Tavazzi G., Dammassa V., Corradi F., Klersy C., Patel B., Pires A.B., Vazir A., Price S. (2020). Correlation Between Echocardiographic and Hemodynamic Variables in Cardiothoracic Intensive Care Unit. J. Cardiothorac. Vasc. Anesth..

[25] Cullen S., Shore D., Redington A. (1995). Characterization of Right Ventricular Diastolic Performance After Complete Repair of Tetralogy of Fallot: Restrictive Physiology Predicts Slow Postoperative Recovery. Circulation.

[26] Mercer-Rosa L., Fogel M.A., Paridon S.M., Rychik J., Yang W., Goldmuntz E. (2018). Revisiting the End-Diastolic Forward Flow (Restrictive Physiology) in Tetralogy of Fallot An Exercise, Echocardiographic, and Magnetic Resonance Study. JACC Cardiovasc. Imaging.

[27] Sade L.E., Gulmez O., Eroglu S., Sezgin A., Muderrisoglu H. (2007). Noninvasive Estimation of Right Ventricular Filling Pressure by Ratio of Early Tricuspid Inflow to Annular Diastolic Velocity in Patients with and Without Recent Cardiac Surgery. J. Am. Soc. Echocardiogr..

[28] Sundereswaran L., Nagueh S.F., Vardan S., Middleton K.J., Zoghbi W.A., Quiñones M.A., Torre-Amione G. (1998). Estimation of Left and Right Ventricular Filling Pressures after Heart Transplantation by Tissue Doppler Imaging. Am. J. Cardiol..

[29] Yogeswaran A., Rako Z.A., Yildiz S., Ghofrani H.A., Seeger W., da Rocha B.B., Gall H., Kremer N.C., Douschan P., Papa S. (2023). Echocardiographic Evaluation of Right Ventricular Diastolic Function in Pulmonary Hypertension. ERJ Open Res..

[30] Fadel B.M., Vriz O., Alassas K., Galzerano D., Alamro B., Mohty D. (2019). Manifestations of Cardiovascular Disorders on Doppler Interrogation of the Hepatic Veins. JACC Cardiovasc. Imaging.

